# 

**DOI:** 10.1192/bjb.2021.62

**Published:** 2022-06

**Authors:** Izaak Lim

**Affiliations:** Adjunct Lecturer, Department of Psychiatry, Monash Medical Centre, Monash University, Clayton, Victoria, Australia. Email: izaak.lim@monash.edu

‘Nature is the future’ reads the design on Elli's sweatshirt, and yet there is nothing natural about Elli or what lies ahead for her. Elli is an android robot, designed to closely resemble a certain 10-year-old girl, who lives with her ‘Papa’ – a man whose real daughter went missing 10 years earlier. Papa keeps Elli in his home as a daughter and sexual companion, and together they reminisce on times when she was alive. So goes the premise of *The Trouble with Being Born*, directed by emerging Austrian filmmaker Sandra Wollner.

Controversial for obvious reasons, the film was withdrawn from the 2020 Melbourne International Film Festival when two forensic psychologists publicly expressed their opposition to its inclusion in the programme. They cited concerns about the film's alleged normalisation of sexual interest in children and its possible exploitation by paedophilic audiences, reigniting a national debate about film censorship. Certainly, the film is ethically challenging and difficult to watch at times, but it has much to offer artistically and psychologically.

*The Trouble with Being Born* is a masterful study of trauma, grief, memory, loneliness and the nature of human (and non-human) relationships. Breathtaking in its complexity and vision, the film explores its disturbing subject matter in a detached (perhaps dissociative) formalistic style, reminiscent of Michael Haneke, Wollner's older compatriot. Far from endorsing the perverse relationship between father/adult and daughter/child, the film is a techno-dystopian parable, warning its audience of the egregious consequences of humanity's attempts to technologically circumvent and transcend the terrible but ordinary vicissitudes of life.

The story pivots when Elli gets lost in the forest surrounding her house and is discovered by a man who gifts her to his elderly mother, Mrs Schikowa. Elli is re-programmed to be Emil – a likeness of Schikowa's brother, who died 60 years earlier. Triggered by reminders of his previous life as Elli, Emil's identity and memory become entangled with Elli's and he becomes increasingly unpredictable, leading us to the film's tragic climax.

Elli and Emil are two ghosts in a machine who haunt the people they left behind, and their unnatural resurrection leads to unnatural consequences. Both Papa and Schikowa are trapped in their grief and guilt – Papa will not confront his loss and keeps himself frozen in the moment of his daughter's disappearance and Schikowa foolishly revisits and attempts to repair her childhood experiences with Emil. Despite its futurism, *The Trouble with Being Born* bears a sense of the archetypal in its exploration of primal anxieties around death, incest and aloneness.

The storytelling in this film is not straightforward and the audience is not left with an answer to the existential question implied by the film's title. Yet the crafted confusion of past and present, and the moral morass we are offered in this film, are signs of Wollner's penetrating insight into the disorder and ambiguity of human nature and experience.

